# Silylated Softwood and Hardwood Lignin: Impact on Thermomechanical and Interfacial Properties of PLA Biocomposites

**DOI:** 10.1021/acs.biomac.5c01179

**Published:** 2025-10-14

**Authors:** Giulia Herbst, Gabriela Adriana Bastida, Quim Tarrés, Marcos Lúcio Corazza, Luiz Pereira Ramos, Marc Delgado-Aguilar

**Affiliations:** † LEPAMAP-PRODIS Research Group, 16738 University of Girona. C/Maria Aurèlia Capmany, 61, 17003 Girona, Spain; ‡ Department of Chemical Engineering, 28122 Federal University of Paraná, Francisco H. Santos Av. 100, 81531-990 Curitiba, Paraná, Brazil

## Abstract

Achieving compatibility between biopolymers and natural fillers is a significant challenge in developing sustainable materials. PLA–lignin biocomposites frequently demonstrate poor interfacial adhesion, mostly due to polarity differences. Softwood (LS) and hardwood (LH) lignins vary in composition and reactivity, affecting PLA structure. This study evaluated the surface compatibilization of LS and LH through silylation at 1, 3, and 5 wt % using a GPS coupling agent. Silylation was validated by TGA, DSC (*T*_g_ increase of ∼3–7 °C), ash color, and EDX (increased silicon). FTIR assessed structural differences in lignins. Rheological tests and melt flow index indicated that unmodified lignin reduced PLA viscosity, while GPS-modified lignin increased it. DSC showed that LS enhanced crystallization more than LH, and GPS at 1 wt % promoted nucleation. Films containing LH at 10 and 1 wt % GPS exhibited improved mechanical properties. Barrier properties remained unchanged, though all films provided UV-blocking capability.

## Graphical abstract

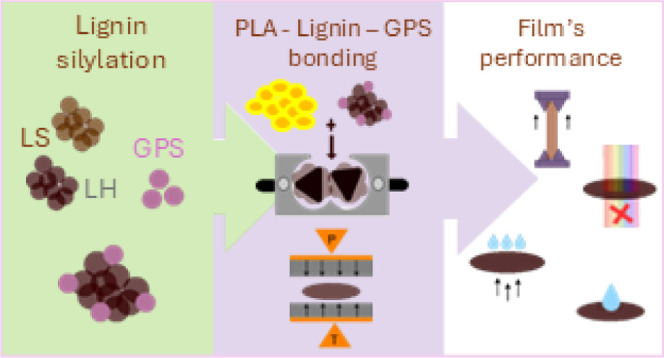

## Introduction

Effective compatibilization remains one of the main challenges in developing functional poly­(lactic acid) (PLA) biocomposites.^1^ Surface compatibilization is critical in determining the performance of polymer biocomposites reinforced with particulate or fibrous fillers.^2^ Effective stress transfer, uniform dispersion, and interfacial stability are dependent on the chemical and physical compatibility between the polymer matrix and the filler surface.^3^ In many cases, inherent differences in polarity, surface energy, or functional group availability lead to poor interfacial adhesion, resulting in phase separation, agglomeration, and compromised mechanical or barrier properties.^4^

PLA offers a renewable platform for a sustainable, competitive alternative to fossil-based polymers, especially for packaging and film applications. Its intrinsic brittleness and low barrier performance limit broader utility.^5^ Lignin, with its aromatic content, antioxidant properties, and UV-blocking properties, has been proposed as a multifunctional additive in the field of food packaging, biomedicine, agriculture, and biomass conversion.^6^ However, as many fillers, its incorporation often results in poor dispersion and phase separation due to limited compatibility with PLA.^7^ Lignin’s chemical heterogeneity, driven by both its botanical origin and extraction process, strongly influences its behavior in polymer matrices, even though its hydroxyl groups are capable of forming numerous hydrogen bonds.^8–10^ In particular, softwood lignins are typically rich in guaiacyl, exhibiting a more recalcitrant structure due to higher quantities of β-5′ and 5–5′ linkages. In contrast, hardwood lignins have a higher syringyl-to-guaiacyl ratio, demonstrating a less recalcitrant and more accessible structure, which affects their polarity, branching, and reactivity.^11–13^ Due to lignins’ distinct structural characteristics, selecting an appropriate lignin type is critical to optimizing polymer–lignin interactions and selecting a satisfactory surface modification strategy.

Lignin acetylation,^14^ esterification,^15^ synthetic chain grafting,^16,17^ and silylation^18^ are techniques that confer lignin polarity or introduce reactive groups that improve interfacial bonding. Silylation has been widely applied due to its ability to introduce organofunctional silane groups that can act as molecular bridges between the lignin surface and the polymer matrix, improving dispersion and adhesion by bonding with available hydroxyl groups present in the lignocellulosic fraction of natural fibers.^19–21^ In previous research, silane coupling agents were successfully grafted onto lignocellulosic fiber surfaces via condensation reactions, improving especially the mechanical properties of PLA biocomposites.^22^

Although several studies have investigated individual modification methods, without systematic comparisons between different lignin sources, particularly in the context of surface-modified PLA biocomposites, remain limited. For instance, Zhu et al. (2015)^23^ studied the effect of three different silane coupling agents at 2 wt % concentration on the silylation of lignin and obtained the maximum tensile strength and Young’s modulus improvement a 5 wt % of lignin, with an increase of around 10% compared to neat PLA. Similarly, Wang et al. (2020)^24^ investigated a technical lignin silylated at multiple concentrations (1, 3, and 5 wt %) in PLA and found minimal improvements in tensile strength, accompanied by persistent agglomeration and low dispersion in both the unmodified and silylated forms.

This study investigates the impact of two chemically distinct technical lignins, softwood (LS) and hardwood lignin (LH), on the structural and functional properties of PLA-based composite films before and after surface modification by silylation. Lignin’s inherent molecular structure, polarity, and functional group variability were approached, and its influence on dispersion, compatibility, and interfacial bonding within the PLA matrix was assessed by film production. This comparative study provides insights into lignin-biopolymer systems through source-specific compatibilization strategies.

## Experimental Section

### Materials

Poly­(lactic acid) pellets (PLA, Ingeo Biopolymer 4043D) purchased from NatureWorks LLC (Plymouth, Minnesota, USA), with a specific gravity of 1.24 g/cm^3^, melt flow index (210 °C/2.16 kg) of 6*g*/10 min, and an average molecular weight of 111 kDa were used as the matrix to produce biocomposites. Kraft softwood and hardwood lignin (LignoForce) were provided by FPInnovations (Pointe-Claire, QC, Canada). The softwood lignin (LS) was produced from the black liquor of a wood mixture of pine (*Pinus contorta*), spruce, and fir at a ratio of 74:25:2, with a molecular weight of 9813 mol/g, and a total OH groups of 6.4 ± 0.4 mmol/g. The hardwood lignin (LH) was produced from the black liquor of aspen wood, with a molecular weight of 4224 mol/g and a total OH groups of 5.8 ± 0.4 mmol/g. Both lignin full characterizations were provided by Suota et al. (2021).^11^ (3-glycidyloxypropyl) trimethoxysilane (GPS), acquired at Merck (Barcelona, Spain), was selected as a coupling agent, aiming at enhancing the interfacial strength of the produced biocomposites.

### Softwood and Hardwood Lignin Silylation Reaction

Lignin was dispersed in water at a consistency of 10 wt % over 700 rpm mechanical agitation at room temperature. Silane coupling agent (GPS) was added dropwise at 1, 3, and 5 wt % concentrations (concerning the dry weight of lignin) and kept under stirring for 30 min. The lignin-dispersed solution was oven-dried at 60 °C until a moisture content of around 4 wt % was achieved. To complete the silylation reaction, the samples were placed for 24 h in an oven at 80 °C before the biocomposites’ melt-compounding.

### Silylated Lignin Characterizations

FTIR-ATR spectroscopy analysis was performed to assess differences in lignin structure and the effect of the coupling agent on unmodified and silylated (1, 3, and 5 wt % of GPS) LS and LH lignin’s chemical composition using a compact spectrometer Alpha II (Bruker Corporation, Massachusetts, United States). Spectra were acquired from a platinum ATR MIR single-reflection accessory at a resolution of 4 cm^–1^, scanned at a wavenumber within the range from 400 to 4000 cm^–1^. Analysis of spectra was performed using OPUS_7.5.18 software.

Thermal analysis was conducted to identify evidence of silylation after GPS addition. Thermogravimetric analysis (TGA) was conducted in a temperature range from 30 to 600 °C at a heating rate of 10 °C min^–1^ under an inert nitrogen atmosphere. Glass transition temperatures (*T*_g_) of the unmodified and modified lignin were obtained from differential scanning calorimetry (DSC, DSC822e, Mettler-Toledo, LLC, USA) and analyzed at a heating/cooling rate of 10 °C min^–1^ from 30 to 210 °C under an inert N_2_ atmosphere. TGA and DSC analyses were performed over both LS and LH lignins, unmodified and modified with a 5 wt % addition of GPS.

The ash content of both unmodified and silylated (containing 1, 3, and 5 wt % of GPS) LS and LH lignin was determined according to TAPPI T211 Ash in wood, pulp, paper, and paperboard.^25^ The samples were ignited in a muffle furnace at 525 ± 25 °C, starting at 250 °C for 1 h, then 350 °C for another hour, and finally the temperature increased to 525 °C until constant weight was achieved.

The elemental composition of the ash residues from both unmodified and silylated lignin (containing 1, 3, and 5 wt % of GPS) was analyzed using Energy Dispersive X-ray Spectroscopy (EDX) coupled with Field Emission Scanning Electron Microscopy (FESEM). The analysis was conducted using a TESCAN Clara scanning electron microscope (TESCAN, Brno-Kohoutovice, Czech Republic), operated under high vacuum conditions at an accelerating voltage of 20 kV. This analysis enabled the quantitative determination of the atomic percentages of key elements present in the ash, and possible effects of surface modification on lignin’s inorganic content and thermal degradation residues.

### Melting-Mixing Composite Preparation and Hot-Pressing Film Production

PLA and lignin were compounded using an internal mixer plastograph (Brabender GmbH & Co. KG, Duisburg, Germany). The equipment consists of a measuring mixer chamber equipped with two counterrotating screws with a processing volume of approximately 55 cm^3^. The raw material was loaded through the top opening into the heated mixer bowl, homogenized by two mixing blades with a roller shape and a rotor speed of 80 rpm, at 195 °C, until a constant torque of the melt compound. Both LS and LH lignins (unmodified and modified) were added at 5, 10, and 15 wt %, respectively. The biocomposites were discharged, cooled down, and milled using a knife mill (SM 100, Retsch GmbH, Haan, Germany) equipped with a 4 mm sieve at the bottom.

Neat PLA and its biocomposites containing unmodified and silylated LS and LH lignin films were produced by hot press molding, in an IDM TEST equipment (Madrid, Spain). Approximately 3 g of each sample, previously oven-dried at 80 °C for 24 h, were placed between high-strength polyester films to facilitate removal from the hot-press metal plates and then molded at 200 °C. The samples were preheated for 60 s, pressed at 45 bar for more than 60 s, and then cooled to room temperature. The resulting film thicknesses were approximately 0.18 ± 0.01 mm for the PLA biocomposites, 0.17 ± 0.02 mm for PLA-LS, and 0.18 ± 0.01 mm for PLA-LH.

### Thermal Properties and Rheological Behavior of the Biocomposites

TGA and DSC analyses of the obtained biocomposites were conducted as described above. Melt flow index (MFI) measurements were conducted in CEAST testing equipment (7082,000, series 17959, Pianezza TO, Italy), according to ISO1133. The extrusion rate was determined as the mass extruded over a specified time under prescribed conditions of 210 °C and a load of 2.16 kg, as described in the datasheet of the PLA applied in this work. The rheological properties, including storage modulus, loss modulus, and complex viscosity of the biocomposites, were measured following the ASTM D4440–15 standards using a modular rheometer (model MCR 302e, Anton Paar GmbH, Graz, Austria). The measurements were taken in oscillatory mode, at the PLA melt state at 190 °C, between a parallel plate geometry arrangement with a gap of 1 mm. The frequency range used was set between 0.1 and 100 Hz. The linear viscoelastic region was determined by an amplitude sweep deformation of 5%, with controlled shear deformation.

### Tensile Properties of PLA/Lignin Films

Tensile properties were conducted using an INSTRON 3340 testing machine equipped with a 100 N load cell. Measurements were performed on strips with dimensions of 15 mm width and 80 mm length at a crosshead speed of 5 mm/min.

### Barrier and Surface Properties of PLA/Lignin Films

The obtained PLA/lignin films’ UV-shielding properties were accessed by a Shimadzu UV-1280 spectrophotometer (Duisburg, European branch office) in the wavelength range 200–800 nm, which was used to measure the transmittance (%) spectra of the specimens. This wavelength range covered both UV and visible light. All samples were cut into a rectangular shape (4 cm × 0.8 cm), placed in a cuvette, measured in triplicate, and used air as reference.

The water vapor transmission rate (WVTR) of the films was estimated by the dry cup method according to the ASTM E-96 standard. The films were cut into circles with a diameter of 6.0 cm and placed in impermeable O-ring cups containing silica gel. The cups were sealed and conditioned at 23 °C with a relative humidity of 50%. After 24 h, the overall weight of the cup, silica gel, and film was measured. The difference was assigned to the moisture uptake of the silica gel, and the WVTR value corresponding to each sample was determined.

The surface hydrophobicity of PLA/lignin films was evaluated by measuring the static water contact angle using a DSSA25 droplet shape analyzer (Krüss GmbH, Germany) equipped with Krüss Advance software. Measurements were performed at room temperature, with data acquisition at a rate of two measurements per second.

### Statistical Analysis

Analysis of variance (ANOVA) and pairwise multiple comparisons with Tukey’s HSD (honestly significant difference) test (*p* < 0.05) were performed using Excel to identify significant differences between LS and LH lignins composition, as well as the impact of the GPS coupling agent on mechanical and barrier properties, including water vapor transmission rate (WVTR) and water contact angle (WCA).

## Results and Discussion

### Effect of GPS Incorporation on Lignin Samples

The structural differences between the LS and LH lignins were investigated by FTIR-ATR analysis, as shown in Figure 1. Typical vibrations of both lignins were shown around 3300 cm^–1^ corresponding to phenolic OH and aliphatic OH groups, followed by vibrations at around 2900 and 2800 cm^–1^ corresponding to methoxyl and methyl. Also, C=O stretch in ketones, aldehydes, and carboxylic acid groups bands in 1700 and 1650 cm^–1^, aromatic skeletal vibrations between 1595 and 1510 cm^–1^, at around 1460 cm^–1^ C–H deformation in −CH_3_ and −CH_2_ groups, and C–C, C–O, C=O stretching at 1225–1274 cm^–1^. Primary alcohol and aliphatic ester C–O stretching were observed in 1085–1030 cm^–1^.^26^ Figure 1A shows LS specific strong vibrations on 1267 cm^–1^ for the guaiacyl ring (G), 1215 cm^–1^ for C–O stretching in G, and at 835 cm^–1^ C–H aromatic bending deformation.^27^ While for LH, Figure 1B, 1325 cm^–1^ vibrations of syringyl ring (S), 1120 cm^–1^ C–H plane deformation of S units, and at 1140 cm^–1^ for G units.^26^ Although silylation did not produce observable changes in either LS or LH for the applied GPS concentration.

**Figure 1:**
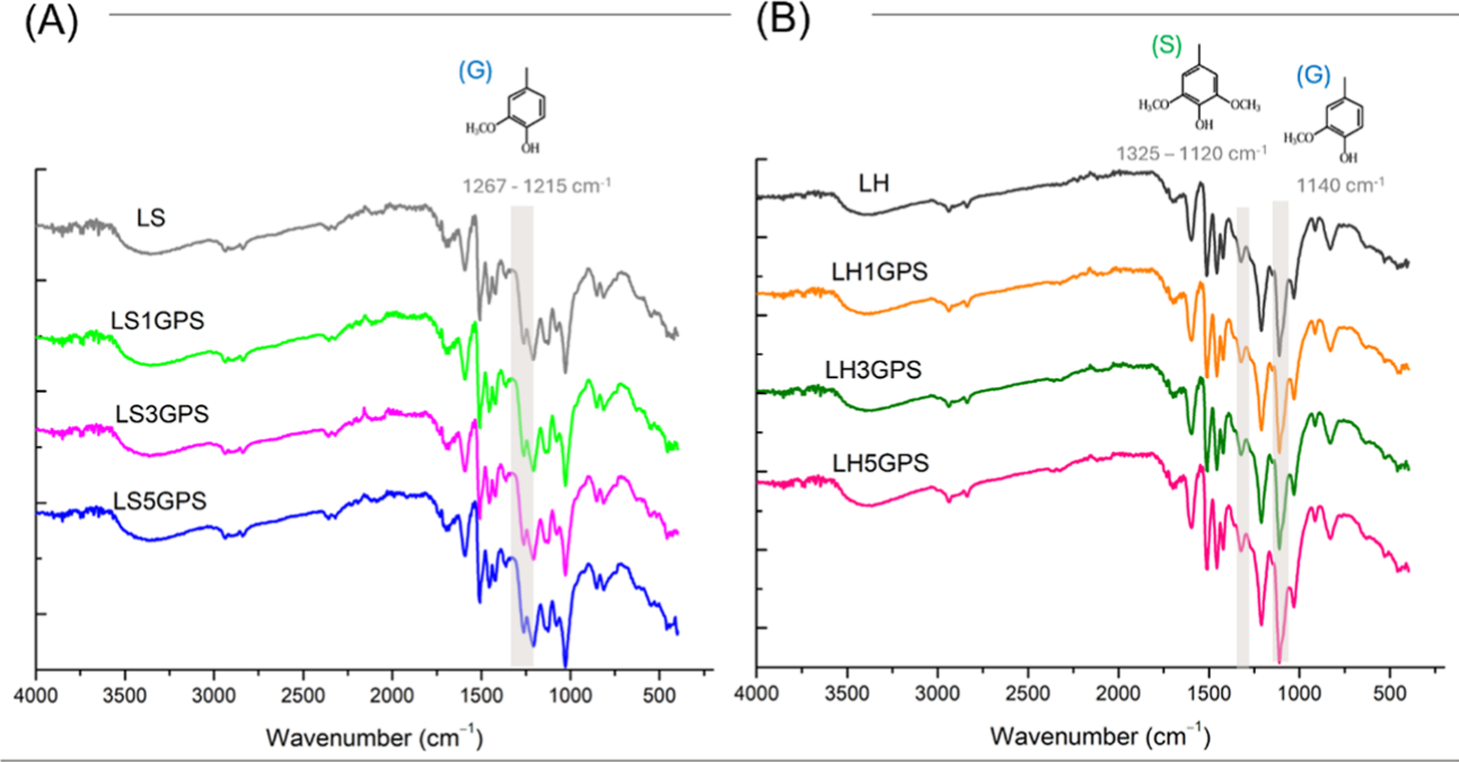
FT-IR spectra of unmodified and silylated soft (A) and hardwood (B) lignin.

The lignin silylation was evaluated by assessing the differences in thermal properties of both LS and LH lignins at a 5 wt % GPS addition and compared to the unmodified ones. Figure 2A shows the degradation profile of both unmodified and silylated lignins in a single step between 30 and 600 °C. Three thermal events were observed, as depicted from the derivative thermogravimetric curve (DTG, Figure 2B), corresponding to moisture release (around 100 °C), followed by the breaking of more labile chemical bonds (between 360 and 380 °C), and the breakage of more stable C–C bonds at temperatures higher than 450 °C. Evaluating the data presented in Table 1, the silylation showed no effect on softwood lignin (LS 5% GPS), maintaining its thermal stability as neat LS. Suota et al. (2021)^11^ characterized both LS and LH technical lignins used in this work and attributed the higher thermal stability of LS lignin to the predominance of guaiacyl units, which tend to undergo condensation at the free C5 position. These reactions lead to the formation of thermally stable carbon–carbon bonds, including 5–5′ and β–β′ bonds. On the other hand, the silylation of hardwood (LH 5% GPS) was able to shift both degradation initial temperatures (*T*_onset_) at 5% and 50% mass loss, promoting an increase of around 30 and 90 °C, respectively, indicating effective cross-linking between LH and the coupling agent GPS.

**Figure 2:**
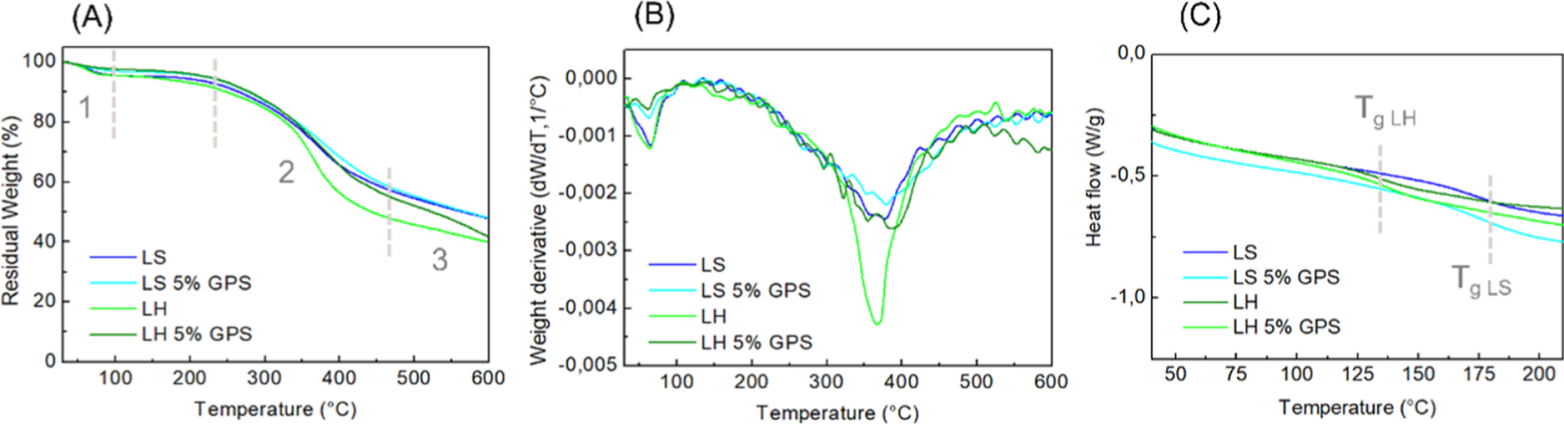
TGA (A), DTG (B), and DSC (C) thermograms of unmodified and silylated soft and hardwood, and lignins.

**Table 1:** Thermal Properties of Unmodified and Silylated LS and LH Lignins

lignin sample	T_onset_ (°C)^a^	*T*_50_ (°C)^b^	residual (%)	DTG_max_	*T*_g_ (°C)^c^
LS	232.3	564.3	48.1	377.0	177.3
LS 5% GPS	233.3	564.3	48.1	378.2	180.5
LH	213.8	433.3	41.7	366.7	132.7
LH 5% GPS	240.8	523.6	40.2	386.6	139.3

a *T*_onset_ = first temperature of degradation registered at 5% mass loss.

b *T*_50_ = 50% mass loss temperature.

c *T*_g_ = glass transition temperature determined by DSC.

The GPS also induced shifts in the lignin *T*_g_, increasing by around 3 and 7 °C for LS 5% GPS and LH 5% GPS, respectively, compared to their unmodified counterparts. Buono et al. (2016)^21^ found that silylation using tert-butyldimethylsilyl chloride (TBDMSCl) led to an increase in *T*_g_. This was attributed to the introduction of bulky silyl groups that restrict the mobility of the lignin chains, thereby raising the *T*_g_.

In order to verify lignin silylation reaction, a simple technique as the ash determination, was applied to evaluate the degree of GPS coupling. Figure 3 shows the variation in the color of ash at varying GPS content. Ash color is essentially related to the inorganic composition of materials.^28^ Unmodified LS showed white coloring due to the higher amounts of calcium in its composition (around 4% of total ash content), while LH presented a light orange coloring due to higher amounts of potassium (around 4% of total ash content).^11^ High concentrations of calcium and magnesium promote the formation of light-colored oxides such as CaO and MgO, typically resulting in white or light gray ash residues. Conversely, high potassium levels often contribute to yellow-orange hues due to the formation of potassium-based salts.^29^ However, the final ash coloration is a complex outcome determined by the combined effects of all the present inorganic elements in the original biomass, including but not limited to iron, which can also impart reddish-brown tones, and manganese, potentially leading to greenish or blackish coloration.^30,31^ The interaction and potential formation of complex compounds among these elements further contribute to the diversity of observed ash colors.

**Figure 3:**
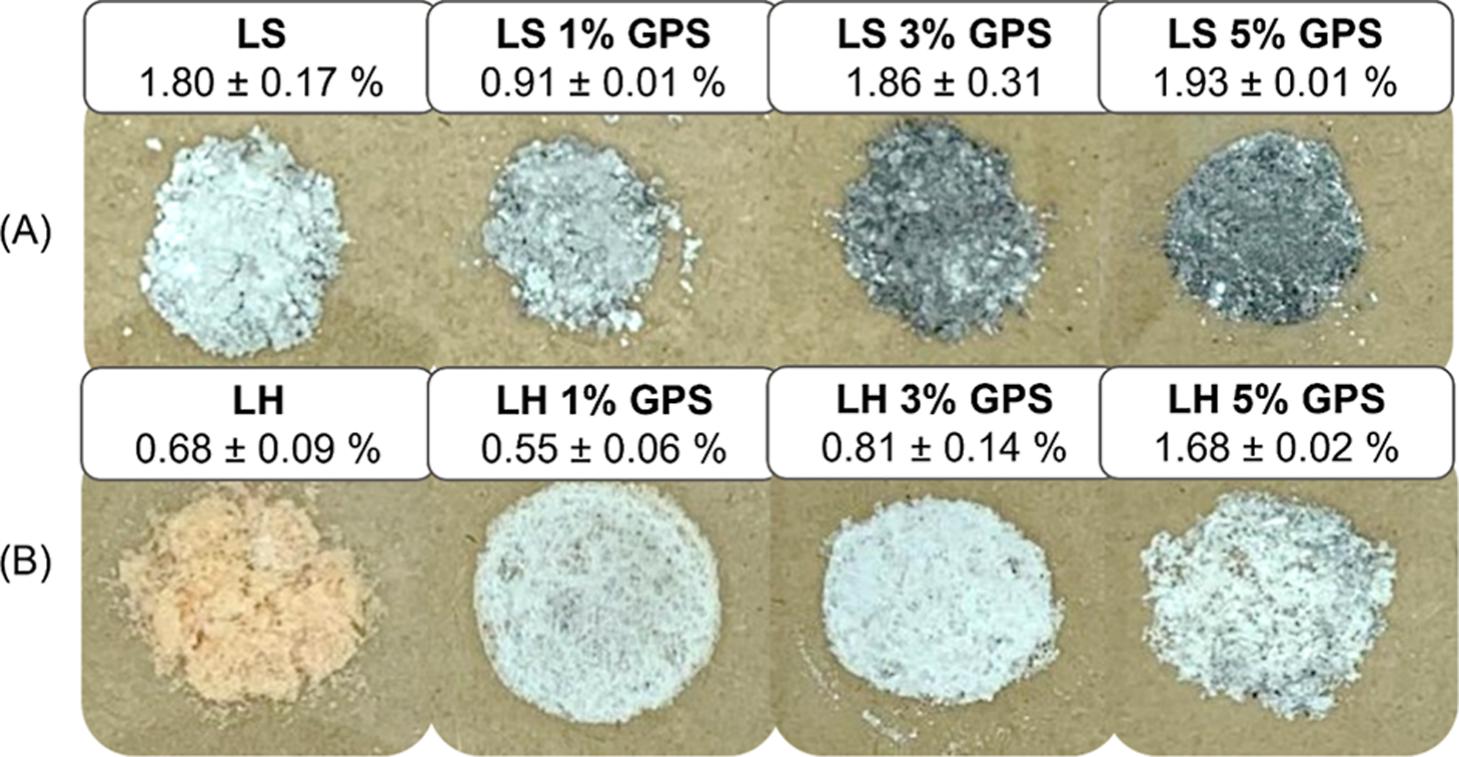
Visual appearance of LS (A) and LH (B) samples after combustion for ash determination at different GPS content.

The silylation degree reflected changes in the material’s thermal decomposition behavior and inorganic composition. As the concentration of the GPS coupling agent increases, the silylation promoted LS coloring turns from light to dark gray. The incorporation of organosilicons alters the combustion process, often resulting in lighter-toned ash due to higher silica content and reduced carbonaceous residues.^28^ Figure 4 shows the ash elemental average composition of the unmodified and silylated lignins. Sodium (Na) and sulfur (S) were the predominant elements found in both lignin ash samples, excluding oxygen (O), and the not showed carbon content of around 40% presented in all the samples as a consequence of the sample preparation. Silicon (Si) was also detected in small amounts, due to its natural absorption by plants.^32^ However, after the silylation reaction with concentrations of 1, 3, and 5% of GPS, the silicon content in the lignin ash increased proportionally, indicating successful grafting and retention of silane moieties.

**Figure 4:**
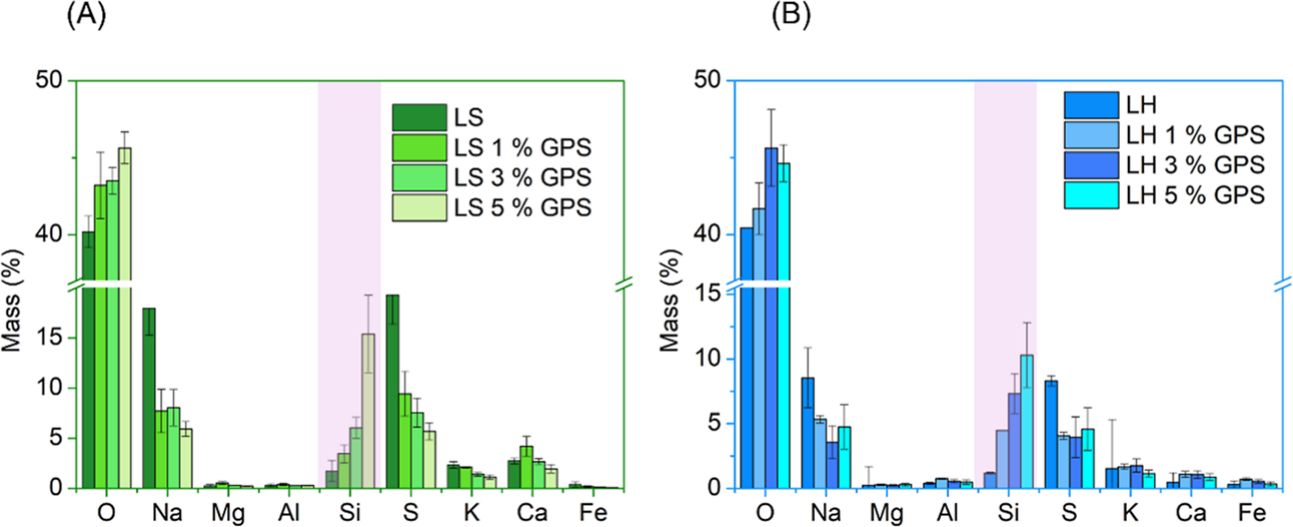
Ash average elemental composition of unmodified and silylated LS (A) and LH (B).

### Thermo-Rheological Properties of PLA/Lignin Biocomposites

The rheological properties of unmodified LS and LH PLA-filled biocomposites compared to the neat matrix at 190 °C and as a function of frequency are shown in Figure 5. Both storage modulus (elastic, *G*′) and loss modulus (viscous, *G*″) slightly decreased in the presence of both LS (Figure 5A) and LH (Figure 5B) lignins, indicating that the composite materials are softer and more compliant, especially at lignin loadings of 5 and 15 wt %. In addition, both the composite materials and neat PLA exhibited predominantly viscous behavior, as indicated by higher *G*″ values compared to G’. This observation is further supported by the loss factor (tan δ) results shown in Figure 5C for softwood lignin, where the biocomposites displayed crossover points (tan δ = *G*′/*G*″ = 1) at higher frequencies, ranging from 50 to 75 Hz, in contrast to neat PLA, which showed a crossover around 40 Hz. This shift toward higher frequencies suggests that the biocomposites maintain a viscous-dominated response over a broader frequency range, possibly due to restricted chain mobility or increased interfacial interactions introduced by lignin.^33^ While for LH, Figure 5D, at 10 wt %, the crossover point (37 Hz) was slightly lower than that of neat PLA, showing a more elastic behavior, suggesting stronger interactions between the hardwood lignin and the PLA matrix.

**Figure 5:**
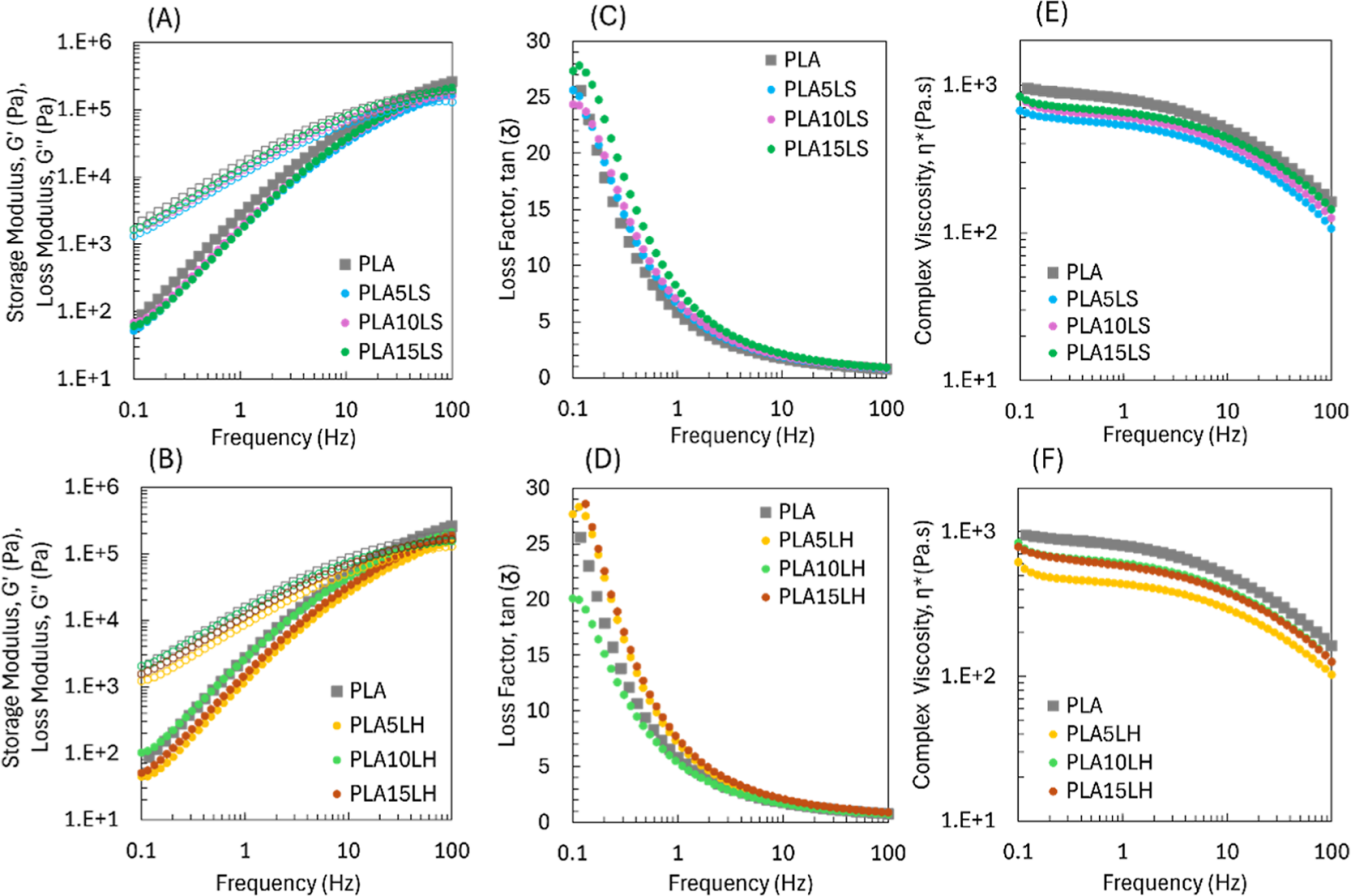
Rheological curves of unmodified softwood and hardwood lignin on PLA.

Moreover, *G*′ and *G*″ showed a linear behavior at low frequencies; consequently, a strong relationship between viscosity and shear rate, evidencing a strong power-law dependence.^34^ The data presented in Table 2 illustrate this phenomenon during the evaluation of the rheological percolation threshold for a uniform dispersion at low frequencies (ranging from 1 to 10 rad/s). The observed reduction in the slope values of *G*′ and *G*″ at a concentration of 10 wt % for both softwood (slope *G*′ and *G*″ of 1.45 and 0.76, respectively) and hardwood (slope *G*′ and *G*″ of 1.53 and 0.84, respectively) indicates improved lignin dispersion through the matrix, and the possible formation of network.

**Table 2:** Low Frequency Slopes of *G*′ and *G*″ for Unmodified and Silylated Lignin Biocomposites

samples	*G*′	*G*″	samples	*G*′	*G*″
PLA	1.60	0.91			
unmodified softwood			unmodified hardwood		
PLA5LS	1.58	0.92	PLA5LS	1.62	0.89
PLA10LS	1.45	0.76	PLA10LS	1.53	0.84
PLA15LS	1.59	0.90	PLA15LS	1.59	0.88
silylated softwood			silylated hardwood		
PLA10LS1GPS	1.54	0.84	PLA10LH1GPS	1.49	0.82
PLA10LS3GPS	1.58	0.86	PLA10LH3GPS	1.55	0.85
PLA10LS5GPS	1.59	0.88	PLA10LH5GPS	1.57	0.86

Regarding the materials’ processability evaluated by complex viscosity (η*), PLA-lignin biocomposites present typical thermoplastics shear-thinning behavior, meaning that with the increase in shear rate, the viscosity decreases.^35^ As observed, the addition of a lignin loading of 5 wt % decreased the viscosity of the biocomposites regardless of the type of lignin that was incorporated, as shown in Figure 5E,F, and slightly increased at loads of 10 and 15 wt %, however, behaving as neat PLA. In this case, lignin acted as a plasticizer agent, requiring less resistance to deformation, showing lower degrees of intercalation, while increasing the free volume of the PLA matrix with the higher loads of lignin.^36,37^ Studies by Ding et al. (2023) also found that the addition of 2.5 and 10 wt % of kraft lignin significantly decreases the composite viscosity at a low shear rate, which was attributed to the lower molecular weight of lignin compared to PLA.^38^

Thus, both LS and LH were modified through silylation reaction to enhance the interfacial adhesion between lignin and PLA, expressed by the low value obtained for storage modulus (*G*′) and complex viscosity arising from polarity and structure differences. The influence of GPS, as a coupling agent, on the rheological properties of PLA-lignin biocomposites was measured at a constant lignin fraction of 10 wt % at 190 °C as a function of frequency (Figure 6).

**Figure 6:**
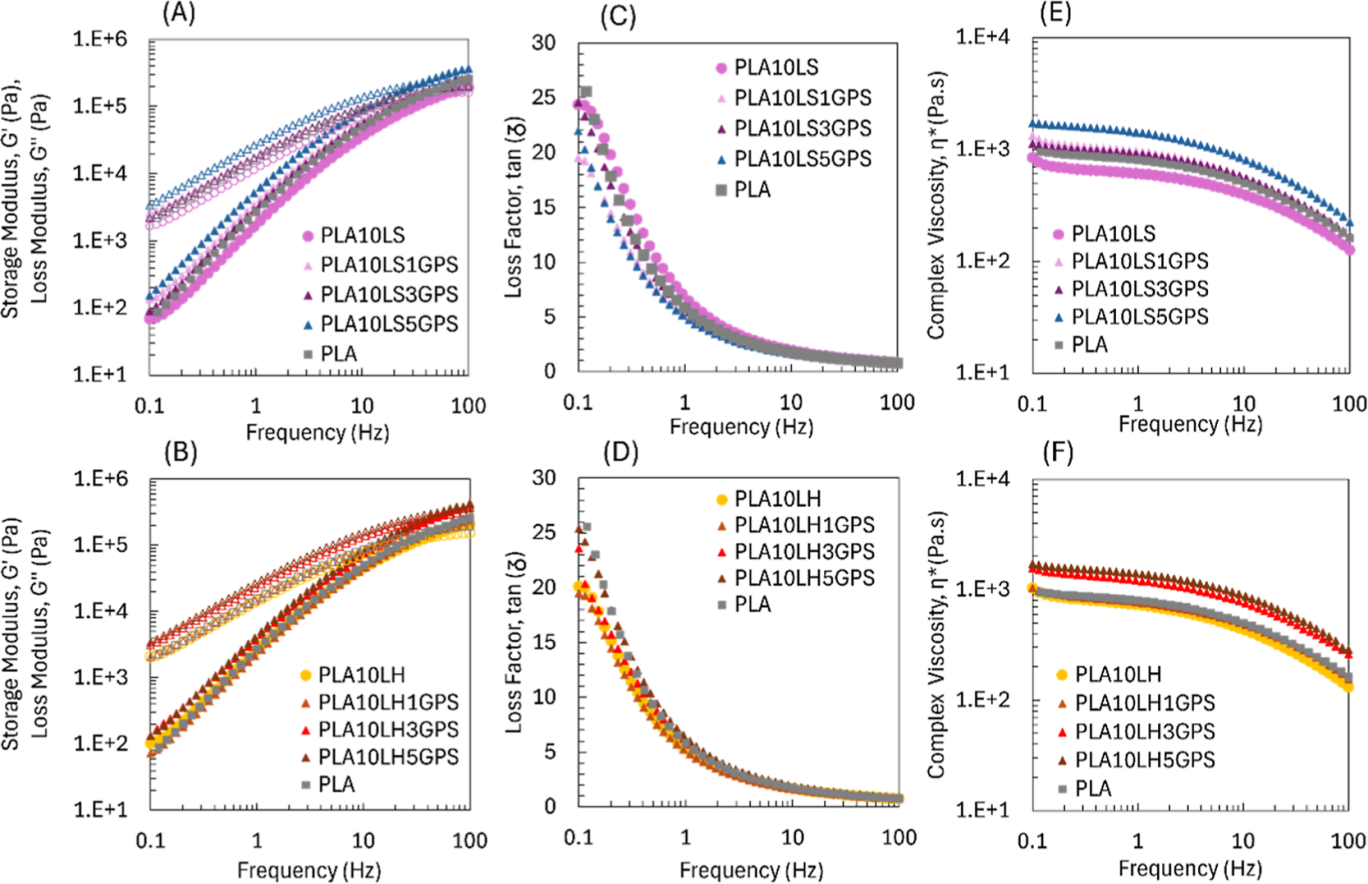
Rheological curves of silylated softwood and hardwood lignin on PLA.

The rheological parameters, represented by *G*′, *G*″, and η*, gradually increased with increasing GPS content in the selected lignins, revealing higher values than neat PLA and the unmodified 10 wt % PLA/lignin biocomposites (Figure 6A,B). Silylation of lignin allowed the formation of localized bonding interactions along the PLA matrix, restricting chain mobility and increasing biocomposite complex viscosity, as well as a significant change in its flow behavior.^35,39^

In previous research, highly fibrillated lignocellulosic fibers, originally from a stone groundwood pulp, were silylated at 3, 5, 7, and 10 wt % GPS contents and incorporated into the PLA.^22^ The lowest degree of silylation (3 wt % of GPS addition) showed the highest enhancement of both *G*′ and *G*″ of the biocomposites due to the covalent bond formation between the available hydroxyl groups and the hydrolyzed forms of GPS silanol groups, while its epoxy terminal groups were able to undergo to open-ring rection with PLA ends during melt processing.^18,22,40,41^ On the one hand, this bond formation creates a strong network between filler and matrix, and possibly a percolation threshold. On the other hand, the excess coupling agent leads to system plasticization or overcompatibilization due to possible GPS self-condensation and steric interference at the filler–matrix interface.^18^

In Table 2, the slopes of *G*′ and *G*″ for silylated lignin biocomposites presented lower values at 1 wt % of GPS content, suggesting optimization of lignin percolation by the coupling agent and better dispersion of both LS and LH up to around 3 wt % of GPS content. This leads to an improved interface and consequently enhances further mechanical properties. However, up to 3 wt %, the slope increased, suggesting weakening of the network, and consequently leading to lower mechanical properties.

The silylation of lignin directly affected the complex viscosity of the biocomposites, as shown in Figure 6E,F. In addition, as the concentration of GPS increased, η* was intensified, reflecting on the biocomposite flowability, as depicted from the MFI values (Figure 7). PLA/LH biocomposites presented higher MFI than PLA/LS (Figure 7A), which is attributed to the higher linearity and condensed structure of LH, contributing to the plasticizing effect and inducing free volume between PLA chains. Something similar was observed with the PLA/LS biocomposites, although the higher complexity of the LS structure and tendency to form aggregates resulted in a decrease in MFI values.^11,42^ The incorporation of the GPS coupling agent led to a significant reduction in the MFI values of both LS and LH biocomposites. In particular, 10 wt % of LH silylated with 5 wt % GPS exhibited an MFI comparable to that of neat PLA. This suggests that lignin modification enhanced interfacial interactions within the molten composite, promoting greater cohesion with the PLA matrix.^43^

**Figure 7:**
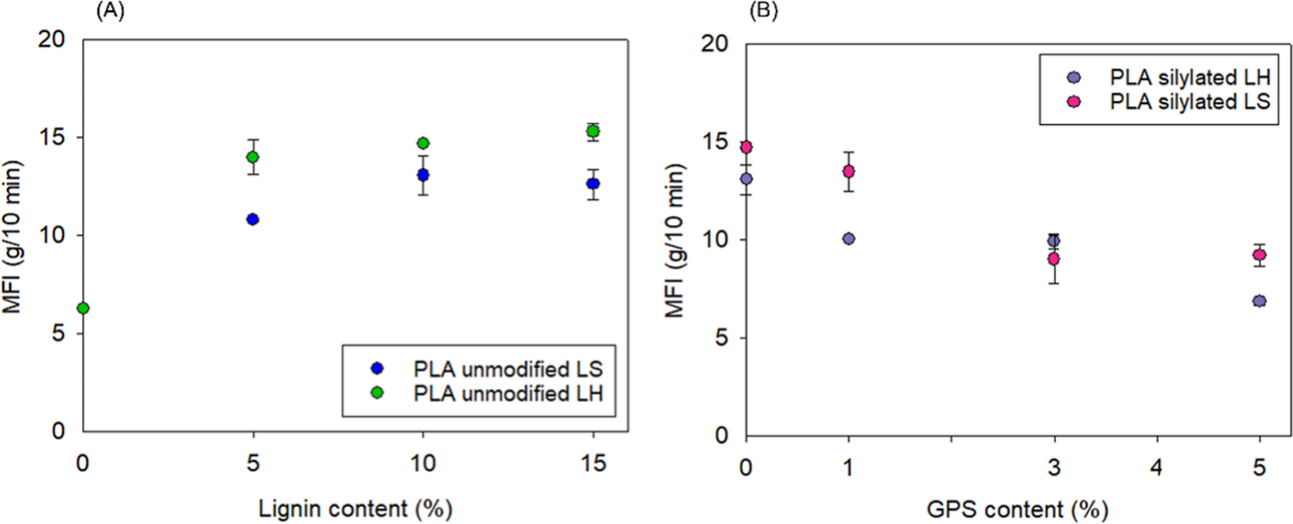
MFI of unmodified and silylated lignin PLA biocomposites.

In summary, the melt rheology indicated that PLA/LS and PLA/LH exhibited lower complex viscosity and higher MFI than neat PLA. Furthermore, lignin silylation introduced interfacial binding sites into the PLA matrix, facilitated by the two distinct reactive groups present in the coupling agent. This modification contributed to the increase in viscosity and may have enhanced the formation of nucleation sites, thus promoting crystallization in the biocomposite.^44,45^ Figure 8 shows the thermal events of unmodified and silylated PLA biocomposites during the second heating scan. Neat PLA presents an amorphous structure after melt-processing, indicating limited recrystallization during the cooling phase. However, the lignin incorporation was able to induce cold crystallization followed by melting, for all the LS compositions, as shown in Figure 8A. The presence of rigid lignin molecules possibly restricts the mobility of PLA chains, limiting their ability to organize and crystallize during cooling. As a result, a higher cold crystallization enthalpy is observed during heating, suggesting a reduced nucleation efficiency in the composite.^46^ Although for LH, Figure 8B, as the amount of LH increases, the cold crystallization and melting events were minimized, due to the amorphous and less condensed nature of this lignin type.^47^

**Figure 8:**
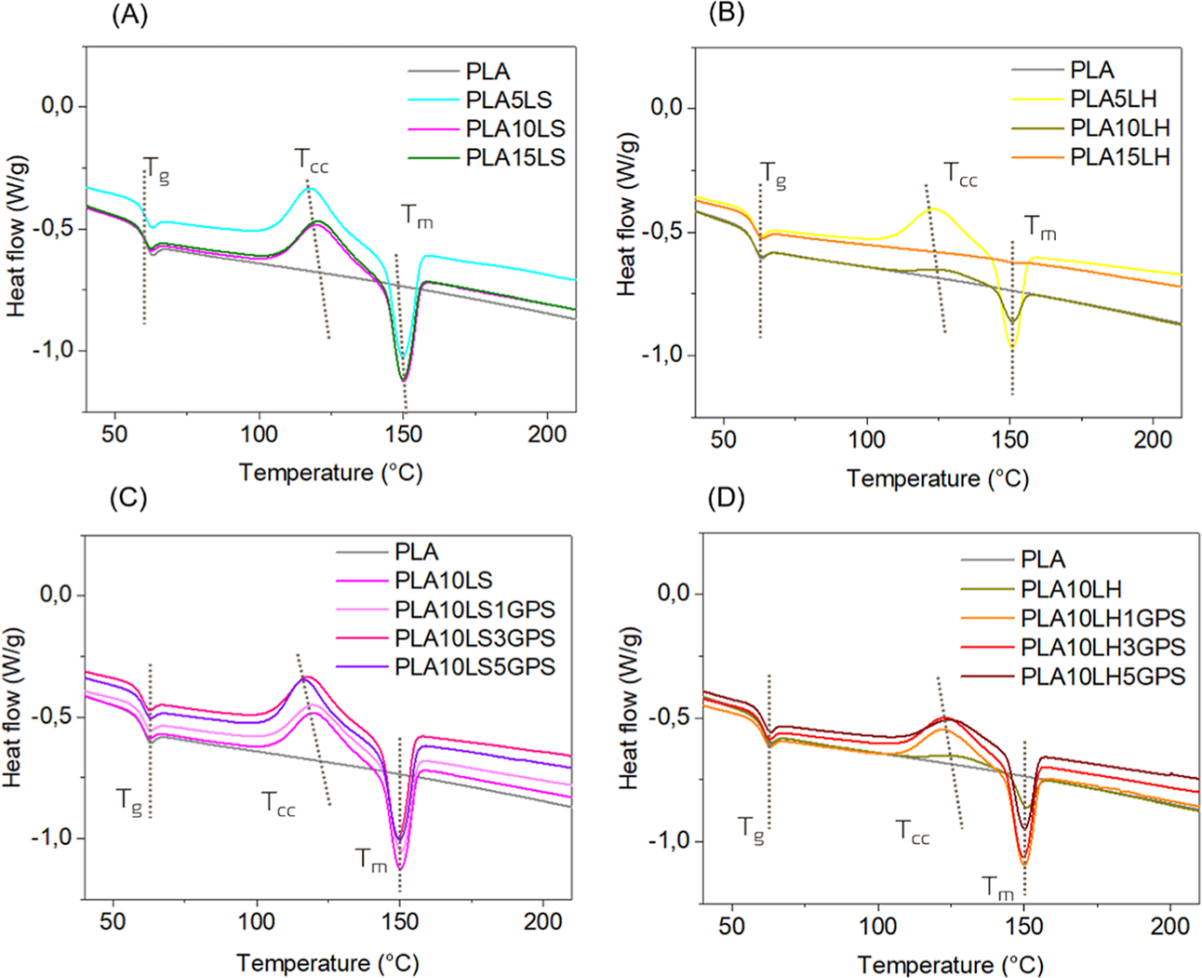
DSC thermograms of unmodified and silylated lignin PLA biocomposites.

After silylation, both cold crystallization and melting events were observed for LS lignin, as shown in Figure 8C. These events were maximized for LH as the GPS concentration increased, compared to the untreated 10 wt % of LH, which is displayed in Figure 8D. The presence of cold crystallization and melting events could lead to an increase in the crystallinity degree of modified lignin PLA biocomposites,^48^ supported by the significant increase in its *G*′ observed in the rheological properties of Figure 6A,B.

The effect of unmodified and silylated lignins on the *T*_g_ and crystallization of PLA is provided in Table 3. All the biocomposites showed a *T*_g_ of around 61 °C, characteristic of neat PLA. The presence of a single *T*_g_ in the DSC thermogram suggests that lignin and PLA are at least partially miscible at the molecular level.^14^ As discussed, the selected PLA presents an amorphous structure after melting, showing no crystallinity index (*X*_c_). Once LS is incorporated at 5, 10, and 15 wt %, the *X*_c_ of the biocomposites increases to around 3%, due to its more condensed structure, which promotes a weak but more effective nucleation of PLA crystals. Despite lignin-induced crystallinity, overall levels remained low since lignin does not act as a strong nucleating agent, in some cases, slowing down or inhibiting the formation of crystalline domains,^49^ as in the case of LH, in which *X*_c_ values range from 0.1 to 1.1%. However, the LS and LH silylation had no clear effect on the PLA crystallization. LS provided the highest *X*_c_ for 3 wt % of GPS, while in the case of LH, it was found at 1 wt % of GPS addition.

**Table 3:** Effect of Lignin Silylation on the Thermal Properties of Softwood and Hardwood PLA Biocomposites

lignin (%)	GPS (%)	*T*_g_ ^a^ (°C)	*T*_cc_ ^b^ (°C)	*T*_m_ ^d^ (°C)	ΔH_cc_ ^c^ (J g^–1^)	ΔH_m_ ^e^ (J g^–1^)	*X*_c_ ^f^ (%)
PLA	0	61.1					0
Softwood							
5	0	61.0	118	149	21.6	24.5	3.2
10	0	60.8	120	150	20.4	22.9	3.0
10	1	61.1	120	150	21.8	24.5	3.2
10	3	60.7	118	149	17.7	21.5	4.5
10	5	61.3	117	150	20.5	21.9	1.6
15	0	60.5	121	150	20.6	22.9	3.0
Hardwood							
5	0	61.0	124	150	17.7	17.8	0.1
10	0	61.3	128	151	4.7	5.6	1.1
10	1	61.0	123	150	18.2	19.5	1.5
10	3	60.8	123	149	18.5	19.4	1.1
10	5	61.1	125	150	14.2	14.4	0.3
15	0	61.3		150	0.0	0.2	0.3

a *T*_g_ = glass transition temperature.

b *T*_cc_ = cold crystallization temperature.

c Δ*H*_cc_ = cold crystallization enthalpy.

d *T*_m_ = melting temperature.

e Δ*H*_m_ = melting enthalpy.

f *X*_c_ = crystallinity index.

### Mechanical Properties of PLA-Lignin Films

The mechanical properties of neat PLA and its biocomposites with lignin derived from unmodified and silylated LS and LH are shown in Table 4. The effect of lignin on PLA is strongly dependent on its composition and structure, which are, in turn, strongly influenced by its botanical origin and extraction process.^50,51^ Lignin derived from softwood species is typically richer in guaiacyl units than hardwood-derived lignin. On the contrary, hardwood lignin, characterized by a higher syringyl content, interacts differently with the PLA matrix and, thus, results in biocomposites with distinct mechanical performance.^37^ Figure 9 shows the evolution of tensile strength at increasing unmodified LS and LH contents, as well as the influence of silylation reaction through the increase of GPS content at lignin weight fraction set at 10 wt %.

**Table 4:** Tensile Properties of Composite Films According to Lignin and Coupling Agent Load^a^

lignin wt %	GPS wt %	tensile strength (σ_t_) MPa	Young modulus (*E*_t_) GPa	elongation (ε) %
0	0	33.4 ± 2.2^a^	2.7 ± 0.1^a^	3.40 ± 0.31^c^
LS				
5	0	35.2 ± 1.7^a^	2.9 ± 0.1^a^	2.04 ± 0.30^a^
10	0	27.8 ± 2.9^b,(a)^	3.2 ± 0.2^a; (a)^	1.45 ± 0.19^b; (a)^
10	1	31.8 ± 1.9^a; (b)^	3.2 ± 0.2 ^(a)^	1.77 ± 0.20 ^(b)^
10	3	23.7 ± 2.1^(c)^	2.7 ± 0.3 ^(b)^	1.92 ± 0.42 ^(b)^
10	5	20.1 ± 2.2 ^(c)^	2.6 ± 0.2 ^(b)^	1.62 ± 0.39 ^(a,b)^
15	0	31.6 ± 1.2^a^	3.0 ± 0.4^a^	1.59 ± 0.27^b^
LH				
5	0	36.4 ± 4.4^a,c; (a,b)^	2.7 ± 0.1^a; (a)^	2.77 ± 0.18^a; (a)^
5	1	38.4 ± 3.2 ^(a)^	2.7 ± 0.3 ^(a)^	2.78 ± 0.42 ^(a)^
5	3	38.6 ± 1.9 ^(a)^	2.9 ± 0.3 ^(a)^	2.56 ± 0.25 ^(a)^
5	5	34.2 ± 2.7 ^(b)^	2.9 ± 0.3 ^(a)^	2.71 ± 0.21 ^(a)^
10	0	40.4 ± 4.8^b; (a)^	2.6 ± 0.1^a; (a)^	2.53 ± 0.35^a; (a)^
10	1	46.4 ± 0.4 ^(b)^	3.0 ± 0.1 ^(b)^	3.10 ± 0.12^c; (b)^
10	3	36.2 ± 2.0 ^(c)^	3.1 ± 0.2 ^(b)^	2.28 ± 0.17 ^(a)^
10	5	33.6 ± 3.2 ^(c)^	3.2 ± 0.2 ^(b)^	2.37 ± 0.30 ^(a)^
15	0	38.2 ± 4.8^b,c; (a)^	3.2 ± 0.2^b; (a)^	2.09 ± 0.19^a; (a)^
15	1	32.1 ± 3.9 ^(a,c)^	2.9 ± 0.2 ^(a)^	2.01 ± 0.17 ^(a)^
15	3	31.8 ± 2.3 ^(c)^	3.1 ± 0.2 ^(a)^	1.80 ± 0.23 ^(a)^
15	5	35.9 ± 2.2 ^(a,b)^	3.1 ± 0.1 ^(a)^	2.04 ± 0.14 ^(a)^

a Superscript letters indicate statistical differences according to Tukey’s HSD test (*p* < 0.05): a, b, and c for comparison between lignin concentrations and (a), (b), and (c) for comparison between GPS concentrations.

**Figure 9:**
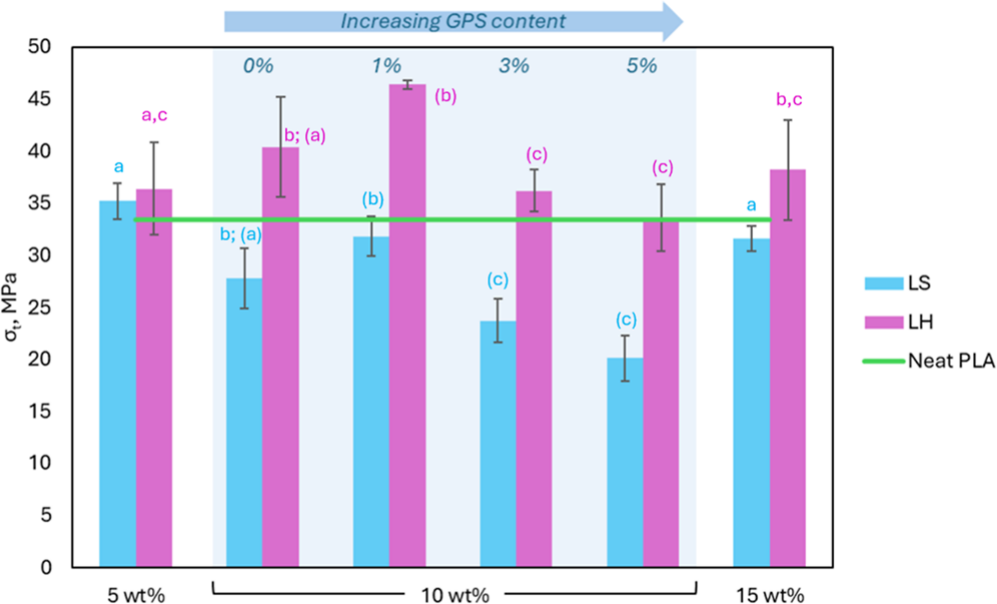
Influence of unmodified and silylated lignin type over tensile strength of different lignin loads, and GPS content of 10 wt % filled biocomposites. Letters indicate statistical differences according to Tukey’s HSD test (*p* < 0.05): a, b, and c for comparison between lignin concentrations and (a), (b), and (c) for comparison between GPS concentrations.

The modest incorporation of 5 wt % of both lignins into PLA resulted in improvements in tensile strength of approximately 5% and 8% for LS and LH, respectively as the lignin content increased to 10 wt %, LH continued to enhance the tensile strength of the composite, achieving an overall improvement of approximately 13%. On the contrary, LS at 10 wt % did not contribute to the tensile strength of the obtained biocomposites, resulting in a reduction of this property when compared to the neat PLA. In this case, reinforcement appears to be driven by mechanical interlocking, and at higher loads, tensile strength decreased due to low compatibility or agglomeration.^52,53^ On the other hand, LH tends to form stronger interfacial bonds, promoting better stress transfer and dispersion within the matrix. Furthermore, the lower glass transition temperature (*T*_g_) of hardwood lignin, approximately 130 °C, compared to softwood (around 177 °C), suggests better thermal compatibility with PLA during melting processing, leading to a more homogeneous mixing and improved interfacial adhesion.^7^

The difference in mechanical behavior can be attributed to the different interactions between each type of lignin and the PLA matrix. LH exhibited a lower content of aliphatic and phenolic hydroxyl (OH) groups, along with a higher methoxy content, reflected in a syringyl/guaiacyl (S/G) ratio of 1.97.^11^ A lower hydroxy group concentration typically results in reduced polarity, which can improve compatibility with the PLA matrix, leading to better dispersion and interfacial adhesion. Furthermore, a higher methoxy content and a higher S/G ratio are associated with a more linear and less condensed lignin structure, which increases chain flexibility and reduces the tendency to aggregation.^54,55^ These characteristics promote uniform dispersion and enable more effective stress transfer within the composite, contributing to improved tensile strength. In contrast, the higher hydroxy group content and the presence of mainly guaiacyl units can lead to stronger lignin–lignin interactions than lignin-PLA, leading to phase separation and poor mechanical performance at higher loads.^37^

Once silylated, the 10 wt % LH biocomposites at 1 wt % GPS showed an improvement of around 13% compared to their unmodified per and around 28% to neat PLA. The attached organosilane groups at the lignin surface increase the dispersion in PLA and improve interfacial adhesion.^23,56^ As a result, the biocomposites based on silylated lignin often exhibit enhanced mechanical performance, especially for LH biocomposites. However, as the GPS content increases to 3 and 5 wt %, the tensile strength of the samples decreases independently of the type of lignin. As observed in the rheological properties, this phenomenon could be related to the excess coupling agent and system plasticization due to possible overcompatibilization, induced by the GPS self-condensation.^18^

Silylated lignins generated an increase in complex viscosity of PLA, as discussed above, indicating enhanced interfacial interactions and possibly restricted chain mobility due to better lignin dispersion or partial cross-linking effects, caused by covalent bonds formed between GPS and lignin during the drying process, anchoring the coupling agent, that upon melt mixing with PLA, GPS can also react with PLA’s carboxyl end groups, strengthening interfacial adhesion and enhancing the composite’s mechanical properties leading to significant improvement in the tensile strength of the LH composite, overcoming neat PLA.^22,41^ The Young’s modulus results in Table 4 for LH biocomposites show higher values compared to neat PLA. At the same time, at 10 wt % of unmodified LH, it was slightly lower, indicating that stiffness was reduced. This suggest that at this lignin concentration, the composite was more flexible and more suitable for film application.^57^ However, after the silylation, the lignin induces a low degree of crystallization (observed by the DSC analysis in Table 3), leading to an increase in Young’s modulus. In contrast, the LS composite showed a decline in tensile strength with the lignin incorporation and after silylation, despite increased viscosity, suggesting that compatibility improvements were insufficient to overcome the inherently lower miscibility or more rigid structure of guaiacyl-rich softwood lignin, leading to an increase in Young’s modulus suggesting that the biocomposites became more brittle, as its elongation at break results also decrease.^58^

### Barrier Properties

Figure 10 shows the visual of the films, and he barrier properties of PLA biocomposites containing unmodified and silylated LS and LH were assessed through ultraviolet light (UV) shielding, water vapor transmission rate (WVTR), and water contact angle (WCA). These properties determine how effectively a film can be applied as a packaging material, protecting contents from external factors such as moisture, extending shelf life, and preserving product quality.^59^ In polymeric films, barrier performance is influenced by the material’s molecular structure, crystallinity, and the presence of additives or fillers,^60^ as in the case of LS and LH.

**Figure 10:**
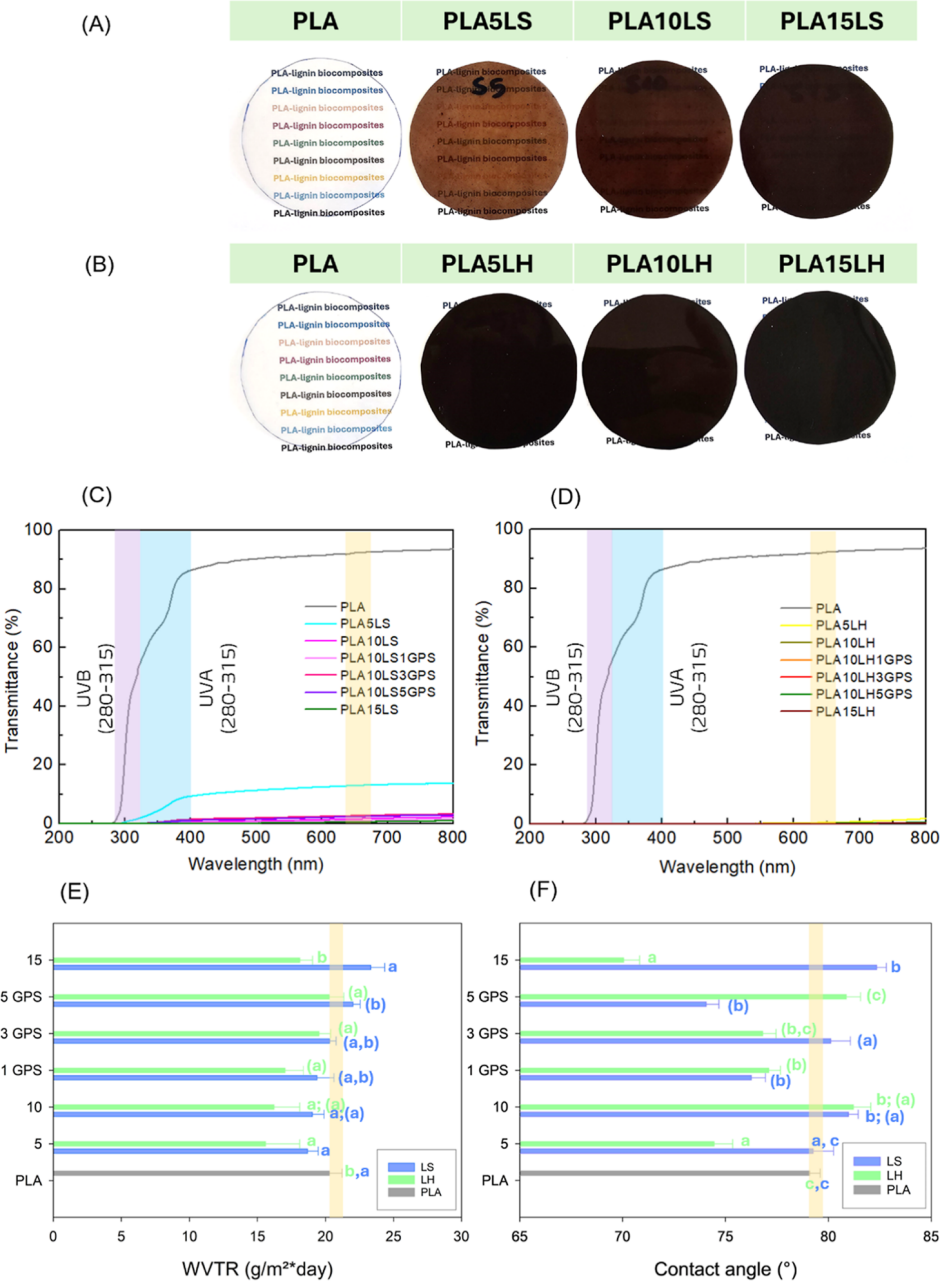
Unmodified and silylated soft and hardwood lignin PLA films’ barrier properties: Photograph of (A) LS and (B) LH films; Transmittance of (C) LS and (B) LH films; (E) Water vapor transmission rate and (F) water contact angle of LS and LH films, in which the letters indicate statistical differences according to Tukey’s HSD test (*p* < 0.05): a, b, and c for comparison between lignin concentrations and (a), (b), and (c) for comparison between GPS concentrations.

Differences in transparency and color intensity with increasing LS and LH content were shown in Figure 10A,B, corroborating the UV-shielding performance of the biocomposites is presented in Figure 10C,D, corresponding to the LS and LH biocomposites, respectively, based on transmittance (%) measurements in the 200–800 nm wavelength range. Neat PLA exhibited high transmittance, ranging from 60% to 85% in the UVA and UVB regions, and approximately 90% transparency at 660 nm, indicating limited UV-blocking capability. Conversely, PLA biocomposites containing 5 wt % LS lignin shows a significant reduction in transmittance, reaching values between 0% and 10% in the UV region and approximately 10% at 660 nm, demonstrating improved UV-shielding capability. As the lignin content increased, the UV-blocking efficiency was enhanced for both LS and LH, attributed to the effective suppression of UV light (transmittance equal to zero). However, this improvement in UV protection suppresses visible light transparency. Lignin exhibits strong UV absorption due to chromophore groups within its complex aromatic structure.^61,62^ Chromophores, such as conjugated aromatic rings, facilitate π–π* electronic transitions, which are primarily responsible for UV absorption. This effect is further enhanced by the functional groups like phenolic hydroxyls, methoxy (−OCH_3_), and carbonyl (−CO), that can interact with the aromatic system, inducing a redshift in the π–π*, intensifying UV absorption across a wider spectral range.^61,63,64^ However, the same chromophore groups are responsible for the characteristic dark brown color of lignin,^65^ which leads to no transparency of PLA-lignin biocomposites.

In addition to UV-shielding properties, the water vapor transmission rate (WVTR) plays a significant role in polymeric films, especially for food packaging applications, and the results are shown in Figure 10 E. Compared to neat PLA, the WVTR of the biocomposites generally slightly decreased as the load of both lignin types increased, due to lignin’s inherently amphiphilic characteristics, presenting both hydrophobic and hydrophilic groups in its structure,^66^ allowing partial compatibility with the PLA matrix. LS displayed values ranging from 18 to 22 g/m^2^·day, while LH showed lower values from 15 to 20 g/m^2^·day. In composite films, micro- and nanofillers act as impermeable obstacles within the PLA matrix, creating a tortuous diffusion pathway that forces water vapor molecules to get cross-dispersed. Reducing the WVTR, thereby improving the barrier performance of the material.^67,68^ Gas barrier properties are often influenced by the crystalline content of the polymer matrix, as crystalline regions are less permeable than amorphous ones.^69^ The DSC results presented in this work (Table 3) indicate that the PLA matrix remained predominantly amorphous. However, the addition of lignin slightly promoted crystallization by introducing a limited number of crystalline domains. These formed crystalline regions may have contributed to a modest reduction in WVTR, although the effect was minor compared to pure PLA. Boarino et al. (2022)^70^ studied the effect of PLA blends containing lignin, lignin nanoparticles, and PLA-grafted lignin nanoparticles with 1, 5, and 10 wt % lignin content in PLA films, and observed a slight reduction in the WVTR as the lignin load was increased, regardless of the lignin form, but especially for the PLA-grafted nanoparticles.

Changes in surface wettability, driven by lignin functional groups, distribution, and structural factors, can also affect the water contact angle of the PLA-lignin biocomposites, as reported in Figure 10F. LS lignin biocomposites showed WCA values ranging from 74° to 82°, with the highest value obtained for 15 wt % of lignin, exceeding the values obtained by LH due to the higher hydrophobic character of LS lignin. Regardless of LS silylation, the sample with 3 wt % GPS exhibited the highest WCA, suggesting that higher GPS loadings may increase moisture uptake, due to unreacted GPS or ungrafted free radicals in lignin or PLA. However, for LH containing biocomposites, corresponding to the one that showed improved mechanical properties, indicating better interactions with the PLA matrix, yielded WCA values ranging from 70° to 82°, with the silylated sample at 1 wt % of GPS showing the highest value. While at 15 wt % of lignin, the value decreased to 70°. Despite the differences between LS and LH, the WCA values only decrease or increase by around 10% of the neat PLA WCA.

## Conclusions

This study demonstrated that both lignin type and silylation significantly affect the thermal, mechanical, and rheological properties of PLA-lignin composite films. FTIR analysis identified structural differences between LS and LH lignins (guaiacyl and syringyl groups), though GPS functional groups were not detected. Silylation increased the *T*_g_ of LS and LH by 3 and 7 °C, respectively, confirming reaction effectiveness. The degree of silylation was visually supported by lignin ash color change: LS from white to dark gray, and LH from light orange to white with increasing GPS content. Rheological analysis showed that LH at 10 wt % and GPS at up to 3 wt % were well dispersed. Both lignin types decreased the complex viscosity (η*) of the biocomposites; however, once silylated, η* increased, suggesting enhanced interactions and network formation within the PLA matrix. MFI values increased from around 6 to 16 g/10 min for both LS and LH, and with the increase of GPS concentration, the values decreased from 6 to 16 g/10 min to 10 wt %. Despite significant changes in the rheology properties, crystallinity increased slightly with GPS (4.5% for LS, 1.5% for LH), with minimal *T*_g_ variation (60.5–61.3 °C). Mechanically, LH-based composites outperformed LS due to their structure and lower molecular weight. LS at 5 wt % reached 35.2 ± 1.7 MPa (statistically similar to neat PLA), while LH at 10 wt % improved tensile strength by around 20% (40.04 ± 4.8 MPa). With 1 wt % GPS, LH composites showed around 40% tensile strength increase (46.4 ± 4 MPa) over neat PLA, confirming enhanced interfacial adhesion. However, silylation had minimal impact on barrier properties (UV blocking, WVTR, and WCA). Overall, the findings highlight the important role of lignin type and surface modification in improving PLA biocomposites for sustainable uses. Future research could explore the effect of shorter-chain silanes and compare the performance of grass lignin with LS and LH to enhance interfacial interactions. Evaluating the long-term stability, biodegradation behavior, and scalability of processing for silylated PLA-lignin systems would also provide valuable insights for future application development.

## Abbreviations

LS: softwood lignin
LH: hardwood lignin
GPS: (3-glycidyloxypropyl) trimethoxy silane coupling agent
